# Brace efficacy: meta-analysis of studies conducted according to the SRS criteria for brace studies

**DOI:** 10.1186/1748-7161-8-S1-O50

**Published:** 2013-06-03

**Authors:** F Zaina, S Donzelli, M Lusini, S Negrini

**Affiliations:** 1 ISICO (Italian Scientific Spine Institute), Milan, Italy; 2 Siena University, Siena, Italy; 3 University of Brescia, Brescia, Italy; 4 IRCCS Don Gnocchi, Milan, Italy

## Background

Bracing efficacy is questioned, since data are very variable, and comparisons are difficult, due to the lack of standard research protocols. The SRS criteria for bracing studies (SRS-C) aimed at comparing different braces, while the SOSORT Management criteria aimed at verifying the quality of brace treatment.

## Aim

To compare the results of studies performed according to SRS-C, and perform a meta-analysis.

## Methods

Design: systematic review and meta-analysis. Inclusion criteria: studies respecting SRS criteria for bracing studies. Protocol: an electronic search was performed in Medline to retrieve all the articles respecting the SRS-C. Data have been pooled, and subgroups made for comparisons. Odds ratios were calculated.

## Results

5 studies have been included (4 retrospective, one prospective), with a total of 416 patients, Cobb Angle range 25-40°, Risser 0-2, more than 10 years old, and less than 1 year post menarche at baseline. Pooling data, we had 40% of patients worsened >36° Cobb, including 27% with curves over 45°; 30% of patients were fused. Making subgroup analysis, we compared rigid braces managed according to SOSORT Criteria (SOSORT-C), with rigid braces managed without: 2% worsened (OR: 95.21; CI 93.75-96.66), without any patients exceeding 45°, or fused, versus 67% worsened (44%>45°Cobb), and 55% fused. Comparing rigid braces altogether with SpineCor, we had similar efficacy, 38% vs 42% worsened (24% vs 31% >45°), 30% vs 29% fused. We had better results for papers respecting SOSORT-C, intermediate for SpineCor, and the worst for the other rigid braces papers with significant OR.

## Conclusion

Pooling data, from studies respecting the SRS-C, showed rate of efficacy that can alter favorably the natural history of AIS, 40% of worsening in high risk patients versus 60-68% described in literature. The SOSORT-C appears fundamental to obtain good results: when they are fulfilled, progression rate is close to zero; when they are not, the efficacy is significantly lower than the one of a soft brace (SpineCor). Bracing is not only a matter of technical efficacy, but also a matter of management. Data from this meta-analysis support the use of braces to change scoliosis natural history, and reduce the rate of surgery.
